# *PERSEUS:* an interactive and intuitive web-based tool for pedigree visualization

**DOI:** 10.1093/bioinformatics/btae060

**Published:** 2024-02-03

**Authors:** Nicole Pradas, Federico Jurado-Ruiz, Carles Onielfa, Pere Arús, Maria José Aranzana

**Affiliations:** Centre for Research in Agricultural Genomics (CRAG), Cerdanyola del Vallès (Bellaterra), 08193 Barcelona, Spain; Centre for Research in Agricultural Genomics (CRAG), Cerdanyola del Vallès (Bellaterra), 08193 Barcelona, Spain; Centre for Research in Agricultural Genomics (CRAG), Cerdanyola del Vallès (Bellaterra), 08193 Barcelona, Spain; Institute of Agrifood Research and Technology (IRTA), Caldes de Montbui, 0814 Barcelona, Spain; Centre for Research in Agricultural Genomics (CRAG), Cerdanyola del Vallès (Bellaterra), 08193 Barcelona, Spain; Institute of Agrifood Research and Technology (IRTA), Caldes de Montbui, 0814 Barcelona, Spain; Centre for Research in Agricultural Genomics (CRAG), Cerdanyola del Vallès (Bellaterra), 08193 Barcelona, Spain; Institute of Agrifood Research and Technology (IRTA), Caldes de Montbui, 0814 Barcelona, Spain

## Abstract

**Summary:**

Pedigree-based analyses’ prime role is to unravel relationships between individuals in breeding programs and germplasms. This is critical information for decoding the genetics underlying main inherited traits of relevance, and unlocking the genotypic variability of a species to carry out genomic selections and predictions. Despite the great interest, current lineage visualizations become quite limiting in terms of public display, exploration, and tracing of traits up to ancestral donors. *PERSEUS* is a user-friendly, intuitive, and interactive web-based tool for pedigree visualizations represented as directed graph networks distributed using a force-repulsion method. The visualizations do not only showcase individual relationships among accessions, but also facilitate a seamless search and download of phenotypic traits along the pedigrees. *PERSEUS* is a promising tool for breeders and scientists, advantageous for evolutionary, genealogy, and diversity analyses among related accessions and species.

**Availability and implementation:**

*PERSEUS* is freely accessible at https://bioinformatics.cragenomica.es/perseus and GitHub code is available at https://github.com/aranzana-lab/PERSEUS.

## Graphical Abstract

**Graphical Abstract:**
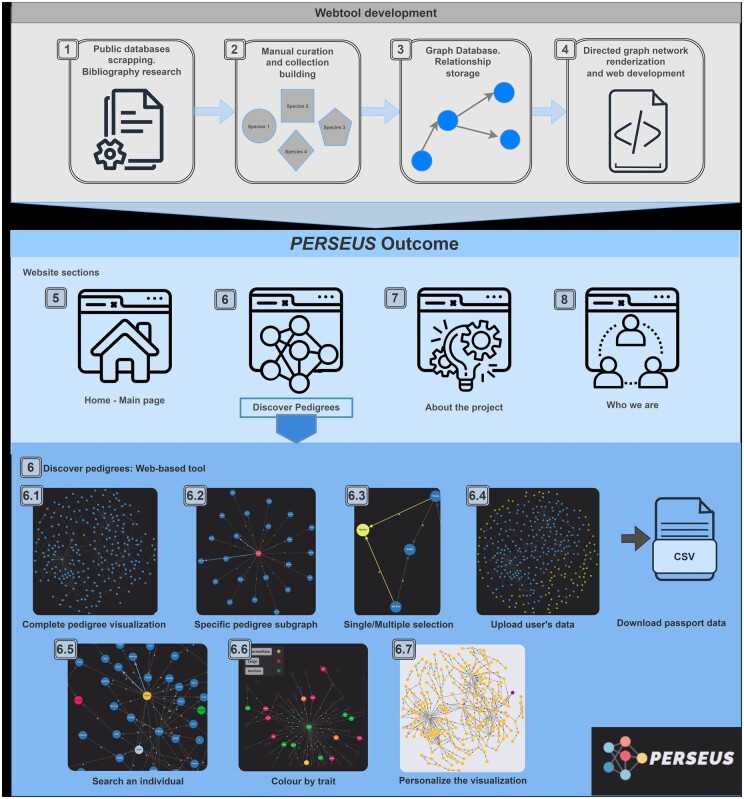

## 1 Introduction

Pedigrees provide insight into the genetic history of an individual and its ancestors, untangling their genetic similarity and predicting the likelihood of offspring inheriting desirable traits (Bérénos *et al.* 2014). Thus, relatedness data can be used to estimate genetic correlations and heritability of certain traits, clarifying the origin of haplotypes shared among individuals with common founders. Ancestry information can also help breeders and germplasm curators to avoid inbreeding by maintaining the genetic diversity within the population and enabling improved management of the germplasm. In addition, it can be used for the characterization and validation of quantitative traits, enhancing the accuracy and efficiency of Marker Assisted Breeding (MAB), haplotype-based analyses (Schaller *et al.* 2023), and genomic prediction/selection strategies (Cobb *et al.* 2019).

Several studies have reported successful pedigree reconstructions from high-quality genotypic data (Hayes 2011, Howard *et al.* 2017, Montanari *et al.* 2020, Muranty *et al.* 2020). The use of molecular markers such as Single Nucleotide Polymorphisms (SNPs) has partially resolved many uncertain or erroneous parent-offspring relationships previously reported. It has also allowed to infer close relationships and to validate historical data in passport information and crossing records, helping to preserve the variability and diversity of breeding materials (Montanari *et al.* 2020, Muranty *et al.* 2020, Schaller *et al.* 2023). As a result, a large number of individuals are described inside pedigree relationships, making the search and visualization of such relationships a challenging goal. To solve this problem, R packages such as Kinship2 (Sinnwell *et al*. 2014), software programs like PEDHUNTER (Agarwala *et al.* 1998), Pelican (Dudbridge *et al.* 2004), CraneFoot (Mäkinen *et al.* 2005), HaploPainter (Thiele and Nürnberg 2005), Graphviz (Zhao 2006), PyPedal (Cole 2007), GeneaQuilts (Bezerianos *et al.* 2010), PedVis (Tuttle *et al.* 2010), Pedimap (Voorrips *et al.* 2012), Helium (Shaw *et al.* 2014), and E-Brida (E-Brida 2018), and web-based tools for family trees like The Pedigree Tool (Lachmund *et al.* 2004) or for crops like maize and soybean such as PedigreeNet (Braun *et al.* 2019) and SoyPedi (Jeong *et al.* 2022) have been already developed for visualizing pedigrees. The majority of these visualization tools have been designed for breeding purposes, reason why some of them require attached databases, perform genetic calculations, and permit the upload of marker allele data. These programs are indeed advantageous when data files can be imported and analyzed for quantitative genetics and genealogy studies. When the final visualizations and analyses are performed, the pedigree displays are mainly presented as images that can be downloaded and stored. Nevertheless, none of these programs or web-based tools offer an open-source, interactive interface in which lineages from previous literature can be searched and their individuals’ information can be looked up, and most of them happen to be available under license. They are mainly based on information uploaded by the user, and do not dispose of a general database of the species. In addition, the vast majority of public cultivar collections and breeding databases only permit the search of one or few individual at a time. This becomes a laborious and time-consuming task when performing multiple queries.

To address the aforesaid limitations, *PERSEUS* has been developed as an interactive web interface used for the storage and public visualization of pedigrees, stored on-site, allowing the searching and filtering for desired features. At this stage, *PERSEUS* contains relatedness and phenotypic data of almond (*Prunus dulcis* Mill.), pear *(Pyrus* genus), apple (*Malus* × *domestica* Borkh), grapevine (*Vitis* genus), and peach [*Prunus persica* (L.) Batsch] crops retrieved from the literature and public cultivar databases and data collections. All this information was rigorously compiled in a unique database which was manually curated. The database was processed, and the parent-offspring relationships were displayed as directed graph networks, distributed using a force-repulsion method. These diagrams are powerful graphical representations to visualize relationships as oriented-point edges (Bostock *et al.* 2011, Have and Jensen 2013). Although *PERSEUS* was initially targeted towards woody crop species, it will be expanded to include other plant or animal species. Hence, *PERSEUS* is thought as a public user-friendly and open-source webtool for breeders, genomic scientists, and agronomists, suitable for exploring parent-offspring and close pedigree relationships and mining relevant information of the individuals of a species.

## 2 Materials and methods

### 2.1 Implementation

*PERSEUS* was developed as a webtool inside a NodeJS runtime environment (Open JS Foundation 2017) in combination with an ExpressJS framework (Open JS Foundation 2023). For visualization of the interactive directed graph networks as SVG elements, the D3 (Data Driven Documents)-based JavaScript v6 library (Bostock *et al.* 2011) was used. The directed graph networks were constructed and distributed alongside the visualization by applying a force-repulsion method among the nodes. JavaScript was chosen for their advantageous implementation outside and across almost all internet browsers. The webtool connects to the open-source graph database Neo4j Software (Huang and Dong 2013), in which the data for the pedigree graph reconstructions are stored. A Python (Python Software Foundation 2023) script was developed to prepare the connection and data storage into Neo4j using the Pandas (McKinney 2010) and NetworkX (Hagberg *et al.* 2008) libraries.

### 2.2 Data used

Pedigree relationships within PERSEUS were gathered from the literatures. The literature search was focused on five woody crop species and their wild relatives for which historical and/or genotypic parent-offspring reconstruction data were reported. In total, 43 publications were selected to feed the database (see Supplementary File S1): 8 for almond, 3 for pear species, 16 for apple, 7 for grapevine species, and 10 for peach. Relationships were either based on genotypic data, some confirmed with reported historical records, or solely in historical data due to lack of a genotype-based pedigree information. In addition, whenever available, information and phenotypic data of the accessions was extracted from the literature, e.g. Malus UNiQue (MUNQ) genotype codes assigned to unique apple genotypic profiles (Denancé *et al.* 2020), country of origin, harvest time, or fruit shape. An automated process of web scraping using Python was employed to gather phenotypic data from public fruit crop databases such as the National Fruit Collection-(NFC) (NFC 2019), the Fruit and Nut Cultivar Database (FNCD) (Main Lab Bioinformatics 2020), and the *Vitis* International Variety Catalogue (*V*IVC) (Maul and Töpfer 2015). Collection, merge, and a manual curation of all the extracted data were performed to summarize in a single database all the information gathered for each species. Inconsistent parent-offspring relationships were discarded to avoid incorrect relationships. In addition, for phenotypic descriptors a simplified list of values was added when >15 observations were available. The complete summarized dataset is available at the Supplementary File S1.

## 3 Web interface

**Figure 1.**
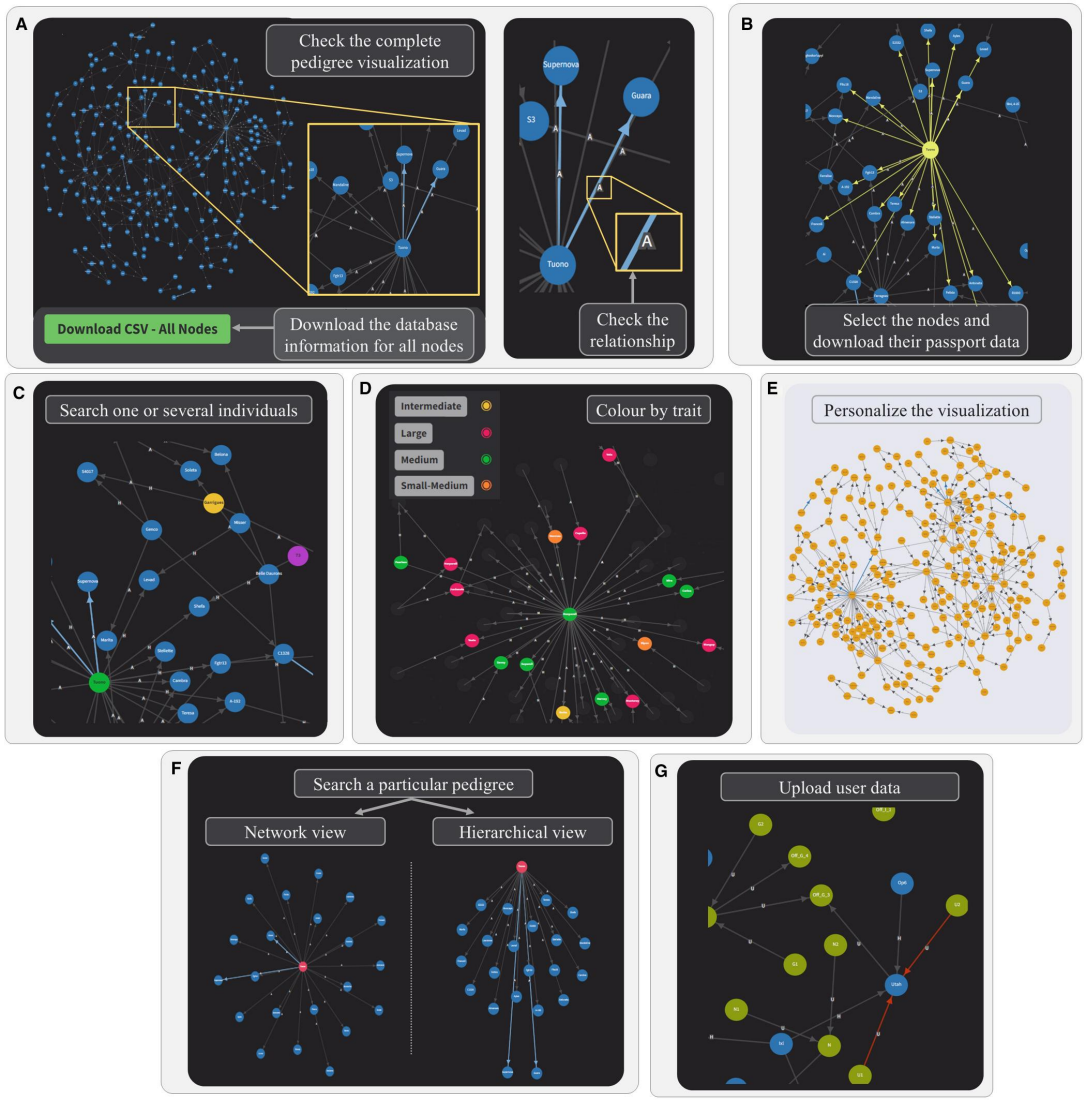
General outlook of the PERSEUS website. (A) The entire pedigree visualization of the species as directed graph networks. Blue nodes represent the accessions/cultivars. The grey edges show the direct Parent-Offspring (PO) relationships, while the blue edges indicate self-fertilization or somatic mutants. On top of each edge, a tag defining the type of relationship appears: (i) H—historical: PO relationship defined in historical data, (ii) G—genotypic: PO relationship obtained though the evaluation of Identity by Descent (IBD) probabilities between accessions based on their genotypic data, (iii) A—absolute: PO relationship both resolved by IBD probabilities and confirmed with historical data. (B) Users can select single or multiple nodes, the selected nodes and all their relationships appear in bright yellow. When clicked, the passport data button appears, and all its stored information can be downloaded. (C) Search for an individual—specific accessions/cultivars can be systematically queried across the complete visualization, chosen and depicted in the visualization. (D) Color by trait—a multi-optional dropdown selector allows to display a set of nodes associated with a particular descriptor accessible in the metadata. (E) Personalize the visualization—the directed graph network visualization can be adjusted as preferred. (F) Search for a specific pedigree—a particular subset of the pedigree is displayed when an individual (appearing in red) and the number of generations (referred to as “distance jumps”) are sought by the user. The subset graph can be visualized as a directed graph network or as a hierarchical tree. (G) The user can upload own data, with the new nodes appearing in green. New relationships in contradiction with the already available ones appear in red as “inconsistent relationships”.

*PERSEUS* allows the search, visualization, interaction, and download of pedigrees. As a first step, users can search for the species of interest and view their complete pedigree (Fig. 1A). The visualization shows a directed graph network, with the blue nodes representing the accessions, and the grey edges indicating direct parent-offspring (PO) relationships. Alternatively, if an offspring was obtained through self-fertilization, the relationship is colored in light blue. The edges are tagged with an H, G, or A, designating if the relationship has been reported with historical data (H), resolved by assessing the Identity By Descent (IBD) probability using genotypic data (G), or both (defined as “Absolute,” A) (Fig. 1A). The complete dataset can be downloaded in CSV format by pressing the “Download CSV—All nodes” button. As a result of the graph’s interactive performance, users can move and select single or multiple nodes. When selected, the nodes and their related edges are colored in bright yellow, and their information stored in the dataset appears on the below section “Selected cultivars” (Fig. 1B). The dataset contains parents and offsprings information alongside with data for traits of interest. Attached to this section, the “Download CSV—Selected Nodes” button retrieves the dataset exclusively containing the selected nodes. Whether the user would like to find a particular individual within the complete graph, the accession name can be searched on the “Search an individual” autocompleting dropdown selection (Fig. 1C). In addition, to easily visualize any attribute associated to a certain trait of interest, users can choose on the “Color by trait” dropdown selection property and the value effects they would like to depict (Fig. 1D). After selecting the trait, a list of the values stored in the dataset is unfolded. The values can then be handpicked, and the individuals characterized with that value are colored. Users are also able to insert their own pedigree data (nodes and/or relationships) in CSV format and merge it with the dataset of interest already available in *PERSEUS.* When user data is uploaded, new nodes are colored in green for their easier tracking, edges are tagged as U (from “User data”), and all the previous functionalities are maintained. In case any novel PO relationship generates inconsistencies with the general data, the edge is colored in red. The entire visualization can also be customized by modifying the size and color of the background, nodes, edges, and tags within the graph by clicking the “Personalize the visualization” selector (Fig. 1E). The pedigree customization is then maintained for all *PERSEUS* functionalities.

Users can opt to visualize only the relationships of a certain accession. The “Look for a specific pedigree” button permits to visualize a subset of the complete pedigree by specifying not only the name of the individual of interest, but also the number of distance jumps (Fig. 1F). Note that the query autocompletes the search with the names available in the dataset. In case a name is not in the dataset, the autocompletion would not return any value. Moreover, the number of distance jumps refers to the number of generations the user can choose to display in the pedigree, starting by the searched individual. For example, a search of the almond “Tuono” with two distance jumps, would return the relationships, if available, up to its grandparent and grandchildren alongside with any of other relationship distanced two jumps, such as the second parent of one of its offspring (Fig. 1F). The specific search is immediately visualized in two different manners: one as a directed graph network view and the second as a hierarchical view. The user can change from one view to the other by clicking the respective “change view” button (Fig. 1E). It is noteworthy that the filtered pedigrees include all the functionalities implemented in the general visualization, also including the option to upload and merge user data. Further description on the use of the webtool can be found on the “About the project” website page. The Graphical Abstract summarizes the webtool development and the *PERSEUS* outcome.

## 4 Discussion and conclusions

*PERSEUS* serves as an interactive and intuitive web interface of public use for breeders, agronomists, and genomic scientists to explore closely related individuals, as well as to search for its relevant traits. Some packages and software have been previously developed to visualize and analyze pedigree data. However, these programs may require a license and/or databases in a complex format which need to be built by the user. In addition, to our knowledge, the desktop apps are limited in their compatibility with different operating systems. As *PERSEUS* runs in a browser, it is compatible with almost any desktop and mobile system. Furthermore, some of these programs lack a user-friendly interface to handpick subsets of relationships among cultivars, the selection of individuals by given traits, or integrating user data with the existing datasets within the tool. Considering these constraints and given the importance of accessing ancestry information to increase crop breeding efficiency, this webtool provides an open-source tool of pedigree reconstruction visualizations that can easier the tracing of published and user’s pedigree data within a unique repository. This can help in the characterization and validation of quantitative traits and assist in the preservation of variability and diversity of breeding materials. So far, *PERSEUS* includes examples of reported pedigrees of five of the most economically important woody crops. As part of its capacity, it also includes phenotypic information mined and curated from public databases and repositories successfully obtained through a combination of an automated web scraping process and manual data mining and curation. Moreover, the interactive network permits an easy display of relationships represented as pointed edges between nodes, having advantageous outlooks at the computational and experimental level. *PERSEUS* can be continuedly updated by adding more parent-offspring relationship data, and additional features such as molecular markers information. Future upgrades of the tool might improve the automated web scraping and manual mining-based methods to extract pedigree documentation from the literature and public databases, which would facilitate the data collection and curation.

## Supplementary Material

btae060_Supplementary_Data

## Data Availability

The data underlying this article are available in its online supplementary material.
